# Wenn ein urologischer Notfall auf eine internistische Krise hinweist: Priapismus als klinische Erstmanifestation einer Leukämie

**DOI:** 10.1007/s00120-020-01326-2

**Published:** 2020-09-16

**Authors:** Muhammad Abdeen, Martin Janssen, Zaid Al-Kailani, Matthias Saar, Stefan Siemer, Michael Stöckle, Gunter Aßmann, Johannes Linxweiler

**Affiliations:** 1 Klinik für Urologie und Kinderurologie, Universität des Saarlandes, Kirrberger Straße, Gebäude 6, 66424 Homburg/Saar, Deutschland; 2 Klinik für Hämatoonkologie (innere Medizin 1), Universität des Saarlandes, Homburg/Saar, Deutschland; 3 Klinik für Urologie und Kinderurologie, Universitätsklinikum Münster, Münster, Deutschland

**Keywords:** Philadelphia-Chromosom, Maligner Priapismus, Hyperviskositätssyndrom, Chronisch-myeloische Leukämie, Fallbericht, Philadelphia chromosome, Malignant priapism, Hyperviscosity syndrome, Chronic myeloid leukemia, Case report

## Abstract

Der Priapismus als klinische Manifestation einer hämatologischen Erkrankung ist selten. In diesem Fall liegt ein sowohl urologischer als auch internistischer Notfall vor, der einer umgehenden Therapie bedarf. Dieser Artikel beschreibt den klinischen Fall eines Priapismus als Erstmanifestation einer bis dahin nicht diagnostizierten chronisch-myeloischen Leukämie (CML) und erläutert die Resultate einer Literaturrecherche zu dieser Thematik.

## Abstract (en)

Priapism as a sign of a severe hematological disease is a rare event, which has to be considered as both a urological and a hematological emergency that requires immediate treatment. This article describes a clinical case of priapism as the first clinical manifestation of a hitherto undiagnosed chronic myeloid leukemia (CML) and discusses the results of a literature review on this topic.

## Falldarstellung

### Anamnese

Ein junger Patient stellt sich in den frühen Morgenstunden notfallmäßig mit einer seit ca. 24 Stunden (h) bestehenden schmerzhaften Dauererektion ambulant vor. Er berichtete von einem ähnlichen Ereignis, bereits 2 Wochen zuvor. Damals habe sich die Erektion nach einer kalten Dusche wieder spontan zurückgebildet. Es bestehen keine Vorerkrankungen oder Voroperationen, insbesondere auch nicht im kleinen Becken. Regelmäßige Medikamenteneinnahme oder Drogenkonsum werden ebenso verneint, wie Traumata oder Geschlechtsverkehr im Vorfeld der Symptomatik. Die weitere Anamnese ergab eine deutliche B‑Symptomatik mit einem Gewichtsverlust von ca. 8 kg innerhalb der letzten 3 Monate, sowie verstärktes nächtliches Schwitzen.

## Klinischer Befund

**Figure 1:**
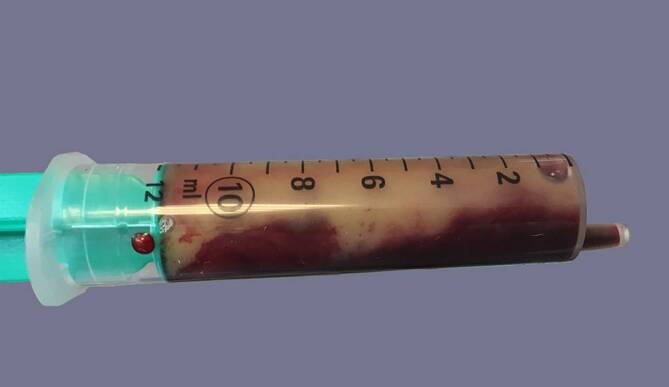

Der 32-jährige Patient ist in gutem Allgemein- und schlankem Ernährungszustand: blasses Hautkolorit, Körpertemperatur 37,2 °C, schmerzhafter, vollständig erigierter Penis bei ansonsten unauffälligem äußerem Genitale. Es erfolgt die problemlose beidseitige Punktion der Corpora cavernosa. Der Versuch aus dem Aspirat eine Blutgasanalyse durchzuführen schlägt jedoch wiederholt aus zunächst unbekannten Gründen fehl. Anschließend werden aus den Schwellkörpern ca. 250 ml Blut aspiriert. Nun fällt während der Aspiration auf, dass sich in den gefüllten Spritzen nach kurzer Zeit eine dicklich weiße Schicht von den übrigen Blutbestandteilen separiert (Abb. 1). Die Laboruntersuchungen des peripher-venös entnommenen Blutes ergeben die in Tab. 1, 2 und 3 aufgeführten Resultate.

**Table 1:** 

Parameter	Wert	Referenzbereich
Leukozyten	422.500/μl	3900–10.200/μl
Erythrozyten	2,76 × 10^6^/μl	4,5–5,9 × 10^6^/μl
Hämoglobin	8,8 g/dl	14–18 g/dl
Hämatokrit	24 %	41–53 %
Thrombozyten	173.000/μl	140.000–400.000/μl
Mittleres Zellvolumen der Erythrozyten	87 fl	80–99 fl
Mittleres Korpuskuläres Hämoglobin	32 pg	27–33 pg
Mittlere korpusukuläre Hämoglobin-Konzentration	37 g/dl	31–37 g/dl
Erythrozytenverteilungsbreite	18,7 %	11,5–14,5 %
Mittleres Thrombozytenvolumen	10,5 fl	7,8–11,0 fl
Unreife Thrombozyten	2,2 %	1,1–6,1 %

**Table 2:** 

Parameter	Wert	Referenzbereich
Natrium	136 mmol/l	135–145 mmol/l
Kalium	4,5 mmol/l	3,5–5–1 mmol/l
Chlorid	96 mmol/l	98–107 mmol/l
Kalzium	2,3 mmol/l	2,2–2,6 mmol/l
Kreatinin	1,20 mg/dl	0,70–1,20 mg/dl
Harnstoff	32 mg/dl	17–48 mg/dl
Harnsäure	8,0 mg/dl	3,4–7,0 mg/dl
Glukose	105 mg/dl	60–100 mg/dl
C‑reaktives Protein	14,8 mg/l	<5,0 mg/l
Laktatdehydrogenase	1.237 U/l	0–262 U/l

**Table 3:** 

Parameter	Wert	Referenzbereich
Neutrophile	80 %	42–77 %
Neutrophile (abs.)	371,3	1500–7700/μl
Basophile	6 %	0–1 %
Esinophile	4 %	0–5 %
Lymphozyten	4 %	25–45 %
Monozyten	6 %	2–10 %
Blasten undifferenziert	2 %	–
Kernschatten	11,8 %	0 %

## Diagnose

Low-flow-Priapismus infolge eines Hyperviskositätssyndroms bei hochgradigem Verdacht auf das Vorliegen einer Leukämie.

## Therapie und Verlauf

In den Schwellkörpern werden jeweils 10 mg Etilefrin mit 10 ml 0,9 % NaCl verdünnt sowie 1000 IE Heparin injiziert. Hierauf sowie unter manueller Kompression kommt es zu einer kompletten Detumeszenz. Es wird abschließend für 3 h ein Druckverband angelegt.

Zur weiteren Diagnostik und Therapie wurde der Patient in die Klinik für Hämatoonkologie aufgenommen. Noch am selben Tag erfolgte eine Beckenkammbiopsie, deren Aufarbeitung letztlich die Diagnose einer chronisch-myeloischen Leukämie (CML) in der chronischen Phase erbrachte. Die molekularzytogenetische Analyse aus den peripheren Blut- und Knochenmarkproben zeigte eine für die CML typische reziproke Translokation zwischen dem langen Arm von Chromosom 9 und dem langen Arm von Chromosom 22 (t[9;22], „Philadelphia-Chromosom“), welche zu einem BCR-ABL1-Rearrangement führt. Zusätzlich ließ sich als sog. Major-route-Zusatzaberration eine Trisomie 8 nachweisen, welche jedoch nicht mit einer schlechteren Prognose assoziiert ist [3]. Die abdominelle sonographische Untersuchung zeigt eine Splenomegalie von 22 × 7 cm, eine signifikante Hepatomegalie liegt nicht vor. Der EUTOS-Score wurde mit 70 (niedriges Risiko) bestimmt.

Nach der initialen Gabe des Zytostatikums Hydroxycarbamid wurde eine Therapie mit dem Tyrosinkinaseinhibitor Imatinib eingeleitet. Hierunter kam es jedoch nur zu einem unzureichenden Ansprechen, weswegen innerhalb von 4 Wochen auf den Zweitgenerationstyrosinkinaseinhibitor Dasatinib gewechselt wurde. Dies führte zu einer Normalisierung des Blutbildes innerhalb von 2 Wochen. Aufgrund der nicht abgeschlossenen Familienplanung wurde vor der Einleitung der zytostatischen Therapie dem Patienten eine Kryokonservierung des eigenen Spermas angeboten. Allerdings lehnte der Patient diese ab. Beim letzten Follow-up des Patienten 22 Monate nach Erstdiagnose ist dieser komplett beschwerdefrei und berichtete von einer normalen Erektionsfähigkeit ohne Rezidiv des Priapismus.

## Diskussion

Als Priapismus wird eine persistierende schmerzhafte Erektion >4 h bezeichnet, die mit der Gefahr ischämisch bedingter Schädigungen verbunden ist und einen urologischen Notfall darstellt. Dieser kann nach Pathogenese und Klinik in einen ischämischem (IP, Low-flow-Priapismus), einen nicht-ischämischem (NP, High-flow-Priapismus) und einen stotternden Priapismus (SP) unterteilt werden [5]. Neben dem idiopathischen Priapismus sowie den „klassischen“ Ursachen (Traumata, Sichelzellanämie, Einnahme von PDE-V-Hemmern, SKAT-Therapie und Psychopharmaka) kann der Priapismus auch Zeichen einer lebensbedrohlichen Systemerkrankung sein. Der maligne Priapismus (MP) ist eine Sonderform des IP, der im Rahmen verschiedener neoplastischer Erkrankungen auftreten kann, so bei diversen soliden aber auch hämatoonkologischen Tumorerkrankungen [2].

Die CML ist eine myeloproliferative Erkrankung, die durch das Vorhandensein der Translokation t(9;22) und der damit einhergehenden Entstehung des *BCR*-*ABL*-Fusionsgens gekennzeichnet ist (das hierdurch verkürzte Chromosom 22 wird auch als „Philadelphia-Chromosom“ bezeichnet). In der Regel erfolgt die Erstvorstellung von CML-Patienten mit laborchemischer Leukozytose, Hepatosplenomegalie sowie unspezifischen Symptomen wie Fieber, Müdigkeit und Gewichtsverlust. In sehr seltenen Fällen entwickelt sich in Folge der Hyperviskosität des Blutes bei der CML ein maligner Priapismus (<3 %) [1]. Noch seltener ist der MP klinische Erstmanifestation der CML. Grundsätzlich beinhaltet das Therapiekonzept bei dieser seltenen klinischen Konstellation die notfallmäßige Behandlung des MP und anschließend die umgehende Initiierung einer Therapie der Grunderkrankung. Nach Anamneseerhebung und klinischer Untersuchung gelten die Punktion der Corpora cavernosa mit Blutgasanalyse des Aspirats und eine Labordiagnostik evtl. inklusive Tumormarkerbestimmung als wichtige initiale diagnostische und therapeutische Schritte beim MP. Führt die Aspiration des Blutes aus den Corpora caveronosa alleine nicht zur Detumeszenz ist die Injektion von vasoaktiven Substanzen (z. B. Phenylephrin) und Heparin angezeigt. Die chirurgische Intervention im Sinne der Anlage einer artifiziellen Fistel zwischen Corpora cavernosa und Glans penis (z. B. Winter-Shunt) ist erst nach Ausschöpfung dieser konservativen Maßnahmen indiziert [7].

Pathophysiologisch entsteht aufgrund der Leukozytose bei der CML eine Hyperviskosität des Blutes, welche zu einer Aggregation von Leukämiezellen im Schwellkörper und konsekutiv zu einem Versagen der physiologischen Detumeszenz führt. Außer der CML wurden die Sichelzellanämie, die chronische lymphatische Leukämie (CLL) und die akute lymphatische Leukämie (ALL) als andere hämatologische Ursachen des Priapismus beschrieben [6].

Obwohl beim IP von 24 h Dauer in den europäischen Leitlinien ein hohes Risiko einer erektilen Dysfunktion beschrieben wird [5], wurde diese in unserem Fall nicht beobachtet.

Diese Arbeit ist nicht die erste, die vom Auftreten eines Priapismus bei CML berichtet. Unser Fallbericht gehört allerdings zu den wenigen, die vom Priapismus als klinischer Erstmanifestation einer CML berichten und dies zudem mit eindrücklichem Bildmaterial untermauern können. Weiterhin gehen wir ausführlich auf die für die Praxis relevanten Therapiealgorithmen ein.

Aufgrund der Seltenheit dieses Falles gibt es für den leukämischen Priapismus keine empfohlene Standarttherapie. Prinzipiell soll die Therapie aus einer lokalen Notfallbehandlung des Priapismus dicht gefolgt von der Einleitung einer systemischen Therapie der CML bestehen. Nach den Leitlinien der American Urological Association (AUA) ist eine systemische Therapie der Grunderkrankung alleine nicht ausreichend, um den CML-Priapismus zu behandeln, eine gleichzeitige intrakavernöse Therapie entsprechend den gängigen Standards der Priapismusbehandlung ist erforderlich [4]. In unserem Fall konnte nach Aspiration, Irrigation mittels NaCl und Injektion von Etilefrin und Heparin eine Detumeszenz rasch erreicht werden. Obwohl Heparin nicht zur Standardlokaltherapie des Priapismus gehört, wurde es hier aufgrund der Hyperviskosität des aspirierten Blutes appliziert.

## Fazit für die Praxis

- Es kann neben den „klassischen“ auch sehr seltene Ursachen eines Priapismus geben, an welche differentialdiagnostisch gedacht werden muss.
- Bei diesen seltenen Fällen kann der Priapismus sogar die klinische Erstmanifestation einer lebensbedrohlichen Erkrankung sein.
